# Mortality in a Cohort of HIV-Infected Children: A 12-Month Outcome of Antiretroviral Therapy in Makurdi, Nigeria

**DOI:** 10.1155/2018/6409134

**Published:** 2018-06-19

**Authors:** Emmanuel Ademola Anigilaje, Sunday Adedeji Aderibigbe

**Affiliations:** ^1^Department of Paediatrics, College of Health Sciences, University of Abuja, Abuja, Nigeria; ^2^Department of Community Medicine, University of Ilorin, Ilorin, Kwara State, Nigeria

## Abstract

**Introduction:**

Recognizing the predictors of mortality among HIV-infected children will allow for concerted management that can reduce HIV-mortality in Nigeria.

**Methodology:**

A retrospective cohort study in children aged 0–15 years, between October 2010 and December 2013, at the Federal Medical Centre, Makurdi, Nigeria. Kaplan–Meier method analysed the cumulative probability of early mortality (EM) occurring at or before 6 months and after 6 months of follow-up (late mortality-LM) on a 12-month antiretroviral therapy (ART). Multivariate Cox proportional regression models were used to test for hazard ratios (HR).

**Results:**

368 children were included in the analysis contributing 81 children per 100 child-years to the 12-month ART follow-up. A significant reduction in EM rates was noted at 17.3 deaths per 100 child-years (30 deaths) to LM rates of 3.0 deaths per 100 child-years (10 deaths), *p* < 0.01. At multivariate analysis, children with a high pretreatment viral load (≥10,000 copies/ml) were found to be at risk of EM (aHR; 18. 089, 95% CI; 2.428–134.77, *p*=0.005). Having severe immunosuppression at/or before 6 months of ART was the predictor of LM (aHR; 17.28, 95% CI; 3.844–77.700, *p* ≤ 0.001).

**Conclusions:**

Although a lower mortality rate is seen at 12 months of ART in our setting, predictors of HIV mortality are having high pretreatment HIV viral load and severe immunosuppression. While primary prevention of HIV infection is paramount, early identification of these predictors among our HIV-infected children for an early ART initiation can reduce further the mortality in our setting. In addition, measures to ensure a good standard of care and retention in care for a sustained virologic suppression cannot be ignored and are hereby underscored.

## 1. Introduction

In 2014, about 58,000 new HIV infections occurred among children in Nigeria, making the country to be the largest harbourer of new childhood HIV infections among the 22 Global Plan Priority Countries committed to curbing HIV epidemics [1, 2]. Unfortunately, only 12% of Nigerian children (aged 0–14 years) living with HIV received antiretroviral therapy (ART) in 2014 [1]. Without ART, half of the HIV-infected children tend to die before their 2nd birthday [3–5]. The proportion of HIV deaths remains higher in resource-limited countries (RLCs) [6–13] compared to resource-rich countries (RRC) [14–16] because of pervasive weak health systems, poor socioeconomic conditions, and a relative suboptimal care for HIV-infected children. The unacceptably high HIV-related mortality in RLCs is also linked to the use of suboptimal antiretroviral regimens, nonadherence to ART, ART discontinuation as a result of drug toxicities, and the propensity for resistant viral strains [17].

Some of the challenges facing paediatric ART expansion in Nigeria and in other RLC include suboptimal provider-initiated counseling and testing in health facilities; problems with early infant diagnosis; inadequate ART centres; shortage of skilled health personnel; lack of appropriate paediatric antiretroviral formulations; and noninvolvement of private health facilities and noncommittal of the government at the local level, to mention just a few [18, 19].

ART is expected to restore immune functions, maintain maximal suppression of viral replications, and reduce HIV-related morbidity and mortality, and improve quality of life, and thus, expected to prolong the survival of HIV-infected individuals [20].

However, in children, a high HIV viremia due to the deleterious impacts of the HIV on the immature thymus and the varying response to ART across paediatric age groups makes the response to ART distinct in children compared to adult populations [21–23].

While few studies [24–26] have reported the outcomes of HIV-infected Nigerian children to ART, only two [24, 26] did an in-depth analysis of baseline factors that may predict mortality. Thus, there is the need for more studies in Nigeria to explore the factors that may predict mortality upon the initiation of children on ART, as this knowledge may allow for close management and/or modification of these predictors of mortality.

This study, therefore, aims at describing the determinants of mortality in a cohort of children that were initiated and followed up on ART for 12 months at the Federal Medical Centre (FMC), Makurdi, Nigeria, between October 2010 and December 2013.

## 2. Methods

### 2.1. Study Area and Setting

This report is from the paediatric HIV care and treatment (PHCT) programme of the FMC, Makurdi, which is supported by the AIDS Prevention Initiative in Nigeria (APIN)/Harvard PEPFAR (The USA President's Emergency Plan for AIDS Relief). The PHCT had commenced a comprehensive care in May 2006. The details of the study setting have been reported elsewhere [27] as the authors had earlier reported the burden of tuberculosis in the same cohort of HIV-infected children.

Two paediatricians and 8 paediatric residents provided the HIV care and treatment in the clinic that is run twice weekly, on Wednesdays and Fridays. Between May 2006 and December 2013, about 1,278 children were already in care, of which 834 had been initiated on ART [27]. ART was commenced on children only if they were confirmed to be HIV-1 infected. Confirmation of HIV infection among infants less than 18 months old required two positive results from HIV DNA/PCR. A double rapid HIV antibody test using Determine HIV 1/2 first and Chembio HIV 1/2 STAT-PAK in a serial algorithm (confirmed with Western blot) denotes HIV infection for children ≥18 months. Children were seen at clinical follow-ups which were scheduled as follows: monthly for the first three months, every 3-month in the first year, and thereafter, every 6-month. During each scheduled follow-up and at any time when children visit the clinic, the following were also noted including the growth parameters, the viral load, the CD4 counts, and comorbidity/opportunistic infection. The flow cytometry method was used for CD4^+^ T lymphocyte (CD4) count measurement, and HIV-1 RNA viral load was also measured.

### 2.2. Ethical Consideration

The Research and Ethics Committee (REC) of the FMC, Makurdi, provided permission for the use of the patients' data for this study. Also, at recruitment of the HIV-infected children in our ART programme, parents or caregivers of these children provided written informed consent for the use of their data for research as would be approved by the REC of FMC, Makurdi.

### 2.3. Study Design and Population

It was a retrospective analysis of available data (sociodemographics, clinical/laboratory, diagnosed comorbidities/opportunistic infections, and mortality) collected among HIV-infected children aged 15 years of age or less, between October 2010 and December 2013. At this period, enrolment into the ART programme is optimal as the initial teething problems related to the programme have been resolved. These issues included technical hitch in viral load (VL) testing procedures, a poor turn-around time for VL results, and poor pharmacy documentation. Excluded from this study were children whose relevant data of interest (as described previously) were missing. HIV-infected children older than 15 years of age were also seen at the adult ART clinic of FMC, Makurdi, and could not meet the population of this study.

### 2.4. Treatment Follow-Up

We have previously described the HIV care and treatment protocol and the follow-up schedule for our HIV-infected children in our earlier publication [27]. In summary, HAART initiation was in accordance with the clinical and age-dependent immunological criteria of the World Health Organization (WHO) guidelines of 2006 and 2010. These guidelines were adopted in the Nigerian *National Guidelines for Paediatric HIV and AIDS Treatment and Care* [28, 29]. Trained nurses and adherence counselor provided counseling on HAART adherence to the children and their caregivers during each clinic visit. Co-trimoxazole (CTX) prophylaxis was provided to all subjects regardless of their CD4 count levels. In addition, children less than 2 years old with HIV-tuberculosis coinfection received HAART regardless of the CD4 count levels. The first-line HAART regimen was zidovudine (AZT) or stavudine (D4T) plus lamivudine (3TC) plus nevirapine (NVP) or efavirenz (EFZ) or lopinavir/ritonavir-LPV/r. The lopinavir/ritonavir-LPV/r was for subjects who had been exposed to NVP in the prevention of mother-to-child transmission of HIV attempts. The first-line HAART (commenced 2–8 weeks after antituberculous therapy) for children weighing less than 10 kg and/or those younger than 3 years of age having HIV-tuberculosis coinfection was a combination of triple nucleoside reverse transcriptase inhibitors (NRTIs) including zidovudine (AZT) plus 3TC plus abacavir (ABC).

AZT or D4T plus 3TC plus EFZ were prescribed for children with tuberculosis (TB) who were ≥10 kg in weight or older than 3 years of age. Children who acquired TB while on 1st-line ART-incident TB (and who were previously on NVP-based regimen and less than 3 years of age or weighs less than 10 kg) had their ART regimen changed to triple NRTIs. If NVP was to be continued in HIV-TB coinfection, it was increased to the maximum dose of 200 mg/m^2^. For subjects ≥3 years or ≥10 kg in weight who developed incident TB, EFV is given or the NVP is substituted with EFV. If the children were on LPV/r-based regimen, the antituberculous regimen would comprise rifabutin instead of rifampicin.

For children to qualify as having treatment failure on 1st-line ART, they must have been on it for at least 24 weeks. For the regimen containing AZT or D4T plus 3TC plus NVP or EFV, the 2nd line regimen comprised ABC or didanosine (DDI) plus 3TC plus LPV/r, and the antituberculous regimen consisted of rifabutin instead of rifampicin. For children who had LPV/r as a 1st line ARV, the 2nd line ART comprised one new NRTI plus 3TC plus NVP or EFV or triple NRTIs. For subjects on triple NRTIs as 1st-line, the 2nd-line regimen contained at least one new NRTI plus 3TC plus NVP or EFV or LPV/r.

Isoniazid (INH), ethambutol (E), rifampicin (RMP), and pyrazinamide (Z) for 2 months, followed by INH and RMP for 4 months (for pulmonary TB) and 7 months (for extrapulmonary TB) was the standard TB treatment regimen. For TB re-treatment, the 8-month treatment regimen consisted of 2 months of streptomycin (S), INH, E, RMP, and Z; 1 month of RMP, INH, E, and Z; and 5 months of RMP, INH, and E.

Both intercurrent and opportunistic infections (OIs) were promptly treated. Ready-to-eat-therapeutic foods (“plumpy nuts”), as well as nutrition education, were administered to those recognized with undernutrition. The caregivers were also encouraged to be part of the HIV support group, especially for children who have not been attending clinics regularly or those being lost to follow-up [30].

### 2.5. Definitions of Cases

Prevalent TB was assumed among children having TB at the time of enrolment into care and before ART initiation.

Incident TB was when children acquired TB while on HAART. It is described as early incident TB-EITB when it occurs within first 6 months of ART. It is late incident TB-LITB when TB acquisition occursafter the first 6 months of a 12-month follow-up period [27].

TB-immune reconstitution inflammatory syndrome (IRIS) was defined as incident TB occurring within the first 6 months of ART in association with good immunological recovery and viral suppression [28, 29].

Virological failure was defined as the HIV RNA becoming reproducibly detectable again after being “undetectable” (i.e., HIV RNA PCR <200 copies/ml) or HIV RNA not suppressed to undetectable levels after 6 months of therapy [28, 29].

The return of the CD4 count to pretherapy baseline or below, in the absence of other concurrent infection to explain the transient CD4 decrease, or a greater than 50% fall from peak levels of therapy of CD4 count in the absence of other concurrent infection to explain the transient CD4 decrease was defined as immunological failure [28, 29].

A high viral load was when HIV RNA copies ≥10,000 copies per millilitre of blood [27, 31].

The WHO age-dependent severe immunosuppression was defined when absolute CD4 count was <1500 cells/mm^3^ in children less than 12 months, < 750 cells/mm^3^ for children 12–35 months, < 350 cells/mm^3^ for children 36–59 months, and < 200 cells/mm^3^ for children ≥59 months [28].

A lost to follow-up (LTFP) was defined as an instance where the child was not seen for three consecutive months from a scheduled visit date.

Anaemia is defined as haemoglobin concentration value less than 10 gram per deciliter [27].

The weight for height Z-score less than −2 standard deviations (SD) (from WHO reference median computed using the WHO Anthro software (version 2.0, 2008) based on the WHO child growth standards of 2006) defined undernutrition in children less than 5 years of age [32].

The body mass index (BMI) (calculated as the weight in kilograms divided by the square of the height in meters (kg/m^2^)) values < 18.5 defined undernutrition in children ≥5 years [33].

### 2.6. Data Extraction

Data were abstracted from the electronic databases and the patients' record files (PRF). Missing data in the PRF were supplemented with data from the electronic databases and vice versa. Data abstracted included those of subjects' age, gender, anthropometric measurements, and other diagnosed comorbidities/opportunistic infections (oropharyngeal candidiasis, diarrheal disease, sepsis, pneumonia, anaemia, and hepatitis B and hepatitis C infections). Time-dependent factors (CD4 counts, the viral load, the haemoglobin value, and the anthropometric growth parameters) were extracted from the Clinical Evaluation Forms at the 6th month of follow-up for each subject. At the 6th and 12th month of follow-up, specific information obtained from the record were those related to prevalent TB, EITB, LITB, vital status state (dead or alive), children lost to follow-up, and children transferred to other health facilities. The cause of death was ascertained using the children's hospital records or by means of a verbal autopsy from family members when deaths were confirmed among children that missed scheduled clinic visit.

### 2.7. Statistical Analysis

The characteristics of the subjects at the commencement of HAART were summarized using means and medians (for continuous variables) and proportions (for categorical variables). Stratification of subjects' ages into 4 groups (<12, 12–35, 36–59, and >59) was done in order to take advantage of the WHO age-dependent immunological grading for HIV-infected children [28]. The main outcome of the study was mortality occurring within the first 6 months (early) and after the first 6 months (late) on the 12 months of HAART follow-up. However, subjects with virological failures were also noted at 6 months and 12 months of HAART. The Kaplan–Meier method was used to assess the cumulative probability of early and late mortality occurring per 100 child-years observation. Subjects that were lost to follow-up, subjects that were transferred out to other ART programme, and subjects that were followed-up to the end of the study (December 2013) were censored for analysis. Associations of baseline factors (sociodemographics, CD4 counts, viral load, undernutrition, and comorbidities) with early mortality were tested for by Cox proportional hazards regression models and expressed for the hazard ratios and 95% confidence intervals. For late mortality, association with some time-dependent baseline factors modified/affected by ART exposure (i.e., the CD4 count, the viral load, the haemoglobin level, undernutrition and opportunistic infections), which were noted at 6 months of HAART follow-up were also tested using the Cox proportional hazards regression models. Factors with *p* values < 0.1 in bivariate analyses were tested at multivariate analyses. For all analyses, confidence intervals (CI) were set at 95% level and *p* values less than 0.05 were considered statistically significant. Statistical analysis was done using the SPSS version 20.

## 3. Results

### 3.1. Baseline Characteristics

Of the 408 children seen during the study period, only 368 were studied. Because of incomplete data, 40 children (9.8%) were excluded from the study. However, these 40 children did not differ significantly in baseline characteristics from the cohort that was studied. The 368 children ranged in age from 0.3 to 13 years. The median age was 5.63 years, and the interquartile range (IQR) was 3–8 years. The majority (57.1%) of the children were 5 years of age and above. There were 206 males (M) and 162 females (F) with a male to female ratio of 1 : 0.8. At the commencement of HAART, the median CD4 count was 623.0 cells/mm^3^ (IQR; 205–1067 cells/mm^3^), and the median viral load (log_10_) was 4.28 copies/ml (IQR; 3.79–5.11 copies/ml). Furthermore, there were 228 (61.9%) children with severe HIV disease (WHO Clinical stages 3 and 4), 213 (57.9%) with viral load ≥ 10,000 copies/ml, 121 (32.9%) children with severe age-dependent immunosuppression (i.e., late presenters), 73 (19.8%) children with tuberculosis (prevalent tuberculosis), and 64 (17.4%) with anaemia at baseline before HAART. There were also 210 (57.1%) undernourished children including 40 (10.9%) under-five children and 170 (46.2%) older children. Adverse reaction was seen in 3 children on EFV but none died.

**Figure 1:**
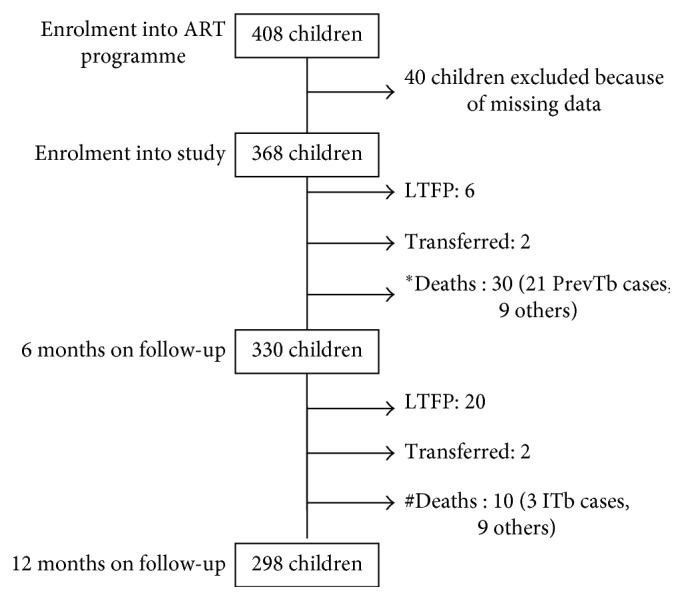
Schematic diagram of the follow-up of the children over 12 months of highly active antiretroviral therapy. ^*∗*^17.3 deaths per 100 child-years; ^#^3.0 deaths per 100 child-years. ART = antiretroviral therapy, PrevTb = prevalent tuberculosis, ITB = Incident tuberculosis, and TFP = lost to follow-up.

Figure 1 is the schematic illustration of the follow-up of the subjects. Among the 368 HIV-infected children that were commenced on HAART, 81 per 100 child-years were followed up to 12 months of therapy. Retention in care and treatment was 89.7% at 6 months and 80.9% at 12 months of follow-up. The median time to HAART initiation was 31 days. There were 40 deaths, with 30 (30/368, 8.2%) occurring in the first 6 months and 10 (10/330, 3.0%) in the later months. There was a significant reduction in mortality rates from 17.3 deaths per 100 child-years at 6 months to 3.0 deaths per 100 child-years at 12 months of HAART (*p* < 0.01). The median time to mortality was 85 days within the first 6 months and 304 days after the first 6 months of HAART.

Table 1 shows the predictors of mortality among the children following 6 months exposure to highly active antiretroviral therapy. In the unadjusted Cox regression analyses, the risk of dying was more in HIV-infected children initiated on EFV-based HAART (cHR; 8.5, 95% CI; 3.360–21.578, *p* ≤ 0.001) or three NRTIs HAART (cHR; 17.6, 95% CI; 7.173–43.069, *p* ≤ 0.001) compared to NVP-based HAART; those with tuberculosis (cHR; 10.4, 95% CI; 4.766–22.745, *p* ≤ 0.001); children with WHO stages 3 and 4 (cHR; 8.7, 95% CI; 2.073–36.531, *p*=0.003); severe immunosuppression (cHR; 218, 95% CI; 6.322–7541.150, *p*=0.003); and those with high viral load (≥10,000 copies/ml) (cHR; 22.2, 95% CI; 3.026–163.111, *p*=0.002). All the 21 patients with tuberculosis that died were diagnosed with the opportunistic infection at HAART initiation (i.e., prevalent tuberculosis). However, in the adjusted Cox regression, only children with high viral load remained a predictor of mortality at 6 months of HAART. The trend was such that the risk of dying in HIV-infected children with a high viral load (≥10,000 copies/ml) was eighteen times more as compared to those with a less viral load (95% CI; 2.428–134.77, *p*=0.005). The risk of dying was not dependent on baseline factors like age, gender, anaemia, and undernutrition.

Table 2 shows the predictors of mortality at 12 months of highly active antiretroviral therapy. In the unadjusted Cox regression analyses, the risk of dying was more in HIV-infected children on LPV/r-based second-line HAART (cHR; 7.9, 95% CI; 2.248–28.284, *p*=0.001) compared to those still on NVP-based HAART, children who developed virological failure (cHR; 9.8, 95% CI; 2.789–35.088, *p* ≤ 0.001), and those with severe immunosuppression (cHR; 5.4, 95% CI; 1.523–19.139, *p*=0.009) on HAART. The risk of death was lower in older children (>5 years of age) with undernutrition (cHR; 0.153, 95% CI; 0.038–0.611, *p*=0.008). However, in the adjusted Cox regression, only those children with severe immunosuppression were at a higher risk of dying. The trend was that the risk of dying while on HAART in the first year of therapy in HIV-infected children with severe immunosuppression at or before 6 months of follow-up was 17 times more as compared to those without severe immunosuppression (95% CI; 3.844–77.700, *p* ≤ 0.001). The risk of dying at the 12 months of ART was not dependent on having or developing tuberculosis even when tuberculosis was seen in some of the children at death. Forty-four children had a virological failure at 6 months of HAART, with a prevalence proportion of 14.3% (i.e., 85.7% had undetectable HIV-RNA copies). Out of these 44 children, 6 (6/44, 13.6%) had died before or at the end of 12 months of HAART. The mean age of the 6 children with virological failure that died was 12 years including 2 girls and 4 boys.

Table 3 illustrates the causes of deaths among the HIV-infected children within the 12 months period of follow-up on HAART. There were 40 deaths within a year of HAART with 30 and 10 deaths occurring within the first 6 months and the later 6 months, respectively. Most of the deaths in the first 6 months of HAART occurred in children with prevalent tuberculosis (21, 70%). Three under-five children who had sepsis and two other children died from severe pneumonia.

At the later 6 months of HAART, severe pneumonia (4, 40%) and incident tuberculosis (3, 30%) were the causes of mortality at the later phase of ART. Two of the four dead children with severe pneumonia were older than 5 years of age and they were also undernourished.

Seven children (4 in the first 6 months and 3 later) died at home with the caregivers only able to give “a history of febrile illness” before their demise.

## 4. Discussion

In this cohort of HIV-infected children that were followed-up for one year, the majority, 89.7% and 80.9% were still on HIV care and treatment at 6 months and 12 months, respectively. The study also revealed a significant reduction in mortality rates from 17.3 deaths per 100 child-years (30 deaths) at 6 months to 3.0 deaths per 100 child-years (10 deaths) at 12 months of HAART.

This pattern of high mortality in the first 6 months followed by a dramatic decrease in death rate agrees with other studies [9, 10], and it suggests that HIV-infected children require some time before the benefits of HAART are fully realized [9, 10]. Bearing in mind the approach to therapy at the time of this study (the intention to start HAART was dependent on clinical and age-dependent immunological criteria), it was impossible to salvage all the HIV-infected children. Furthermore, the number of such HIV's mortality may not decrease with HAART being initiated much later in the disease course. Similar trend was also noted by other researchers [9, 10].

In multivariate Cox regression analyses, mortality at 6 months of HAART in our cohort was dependent on having a high pretreatment viral load (>10,000 copies/ml), with the risk of dying being 18 times more; while, HIV-infected children still having severe immunosuppression at/or before 6 months of ART are 17 times more likely to die before the 12 months of HAART.

The mortality rate of 3.0 deaths per 100 child-years in this study is lower than the rates of 4.0, 4.7, and 8.4 per 100 child-years reported in Ethiopia [7], South Africa [8], and Kenya [6]; but it is higher than the rates in other Nigerian studies which reported 1.0 death per 100 child-years and 2.67 deaths per 100 child-years in Jos [24] and Lagos, respectively [26].

Outside Africa, the mortality rate of 3.0 deaths per 100 child-years in this study is higher than the 2.0 reported in the United Kingdom [16] and the 2.31 per 100-child years in China [34].

While studies in Africa on HIV-related mortality are complex because of other significant non-HIV causes of mortality [3–11, 24], we suggest the following factors to be responsible for the relative good survival in the present cohort who presented mostly (61.9%) with severe HIV disease (WHO stages 3 or 4) and (57.9%) with viral load ≥10,000 copies/ml.

Firstly, the good training and retraining on paediatric HIV care and treatment received by the healthcare providers caring for these children from the Harvard PEPFAR programme may have impacted positively on the supportive care given to these children. Secondly, a robust ART adherence support group (i.e., the kiddies' club) in our ART programme which engages parents/caregivers in ensuring an optimal ART adherence among the children [30]. Thirdly, the fact that all the children less than 24 months of life were commenced on HAART regardless of their CD4^+^ cell counts, in line with the Nigerian Guidelines of paediatric ART of 2010 [29]. The fifth reason is that all the HIV-infected children in this cohort were placed on CTX prophylaxis regardless of their CD4 counts. The role of CTX prophylaxis in reducing morbidity and mortality in HIV-infected children has been confirmed by other studies [24, 35]. And lastly, the children commenced HAART at a relatively high median CD4 count of 623.0 cells/mm^3^ compared to the Jos [24], South African [8] and Kenyan [6] studies with a lower baseline median CD4 counts of 480 cells/mm^3^, 226 cells/mm^3^, and 286 cells/mm^3^, respectively.

In this study, having a high viral load (≥10,000 copies/ml) at baseline increases the risk of dying in the early phase of HAART by 18 times compared to when individuals start HAART at lower values. This increased risk of death with a high viral load exists regardless of the types of HAART regimen, whether the children were infected with tuberculosis or not, the WHO clinical stage, and the degree of immunosuppression, all of which appear to impact on mortality at an unadjusted Cox regression analysis.

Specifically, in the study by Wang et al. among Chinese adults (16–80 years old), patients having VL ≥ 10,000 copies/ml and HIV-drug resistance were two times as likely to die compared to patients with VL < 1,000 copies/ml [36]. Furthermore, individuals with VL ≥ 10,000 copies/ml and without HIV-drug resistance were also 1.7 times as likely to die [36]. Although we did not test for HIV resistance in our cohorts with a high pretreatment viral load, HIV-drug resistance in ART-naïve individuals with high viral load was suggested as the reason for the increased mortality in the study of Wang et al. [36]. While Hogg et al. [37] and Zaccarelli et al. [38] also reported a high mortality in their patients with drug-resistant HIV infection with a high viral load, two other studies did not find same [39, 40].

Boosted protease inhibitors (Boosted PI) are often used in 2nd line HAART regimen because they are associated with multiple clinical benefits, including higher efficacy against resistant HIV strains [41] and a lack of PI-associated resistance mutations in treatment-naïve patients [42]; unexpectedly, our study revealed that at unadjusted Cox regression, our children on LPV/r-based second-line HAART tend to die more when compared to those still on NVP-based HAART at 6 months of therapy. We suggest that the children that died on LPV/r were commenced rather late on it (mean age at death was 12 years), had developed resistance to it, or may have had a suboptimal adherence to it.

Within the first 6 months of ART, children on EFV-based or three-NRTI HAART compared to NVP-based HAART, those coinfected with tuberculosis, children with severe HIV disease (WHO stages 3 and 4), and children with severe immunosuppression are also associated with higher mortality, albeit at unadjusted Cox analyses. After the first 6 months, the risk of dying was also more in children who developed virological failure, also at unadjusted Cox analyses.

Worthy to note is the fact that all the children who died on EFV-based HAART and three-NRTI HAART were also coinfected with tuberculosis.

Similar trends in mortality with HIV/tuberculosis coinfection, severe HIV disease (WHO stages 3 and 4), virological failure, and severe immunosuppression have been noted in numerous studies [43–47], thereby providing a focus for targeted management of high-risk HIV-infected children at risk of dying [47].

In this study, the risk of dying while on HAART in the first year of therapy in children with severe immunosuppression was 17 times more when compared to those without severe immunosuppression. In other words, HIV-infected children who fail to achieve CD4^+^ T cells recovery at 6 months of ART are at risk of dying before and or at 12 months of HAART. Thus, even in advanced HIV disease or in children with virological failure, the reconstitution of the immune system induced by HAART is due to an increase in the activation of the naive CD4^+^ T cell of thymic origin [48]. This immune system restoration thereafter supports the normalization of immune parameters (cytokine production, lymphoproliferative response, and immunoglobulin levels in plasma), with the recovery of naive CD4^+^ T cells occurring more rapidly if treatment is started at a younger age [48]. When CD4^+^ T recovery does not happen, associated comorbidity from opportunistic infections like tuberculosis and life-threatening bacterial infections contribute to mortality [24].

As most of the children that died in this cohort had a dual HIV-tuberculosis coinfections (21/30, 70% of deaths in the first 6 months; 3/10, 30% in later months), tuberculosis was not found to be associated with mortality at multivariate Cox analysis. However, HIV-infected children had been known to demonstrate greater mortality from tuberculosis, with mortality as high as 20–35% in RLC [49–51]. In actual fact, all children who have dual HIV-tuberculosis infections at the time of their deaths are internationally classified as having died from HIV [52].

Severe undernutrition in HIV-infected children usually results in marked depression of both cell-mediated and humoral immunity reducing their ability to cope with life-threatening infections [30, 31]. In addition, micronutrient deficiencies may decrease the effectiveness of ART among undernourished individuals [53].

Neither undernutrition nor anaemia is a predictor of mortality in this study in contrast to what was reported in other studies [6, 9, 10, 54–56]. Incidentally, the risk of death was lower in older undernourished children (>5 years of age) among whom two of the four dead children also had severe pneumonia. Bacterial infections, pneumonia, and tuberculosis were the main causes of mortality in this study. A similar trend had been observed by two previous studies [6, 14]. While the causes of death in children that died outside the hospital were unknown, verbal autopsy from their caregivers was that of a “brief febrile illness” also, in keeping with a possible infectious cause.

The risk of death also did not depend on age or gender in this study. This contrasts with the study by Ebonyi et al. [24] whereby the risk of dying was about three and half times more in children <5 years compared to those >5 years. Also, in the study by Bolton-Moore et al. in Zambia, age was found to be a determinant of mortality as the median age of children on ART who died was 46 months [9]. In South Africa, Zanoni et al. also reported that female gender was associated with an increased risk of HIV-mortality in children initiated on ART [8]. In a North American study, Foca et al. also reported a higher mortality in female HIV-infected children [57]. However, Zanoni et al. concluded by saying that they were unsure if the gender-related HIV-mortality was as a result of biological differences between the genders [8]. Reasons put forward to explain the higher mortality in female gender included gender-related discriminations that make parents delay bringing female children to care, as parents pay more societal values on their male children in South Africa [8].

## 5. Limitations of the Study

Being a retrospective study, the small sample size may have reduced the precision and the observed effect sizes of some variables (i.e., HAART regimens, tuberculosis, WHO clinical stage, virological failure, and undernutrition) in detecting a difference at multivariate Cox regression analyses. In addition, the small events (30 and 10 deaths before, and after 6 months of HAART) may have limited the power of assessing the significance of aforementioned variables in multivariate analyses.

Also, adherence to ART is an important factor in treatment success, but we assessed adherence using self-report information from the caregivers. The reliability for accurate true adherence is therefore limited.

It is also conceivable that some children that were lost to follow-up had actually died, which might have affected both the validity and precision of our estimate as pointed out by an earlier systemic analysis done in 2009 [58]. However, a recent systemic review in 2017 suggests that more of sub-Saharan HIV-infected patients that are lost to follow-up are surviving better [59]. Also, the lack of postmortem studies in our program and the reliance on the verbal autopsy for children that died at home may have led to an inaccurate labeling of the cause of death in such cases. We also could not do genotypic resistance testing among our children with virological failure at the time the study was conducted.

Regardless of these limitations, this study is strengthened by the fact that time-dependent baseline factors modified/affected by ART exposure (i.e., the CD4 count, the viral load, the haemoglobin level, under-nutrition, and opportunistic infections) were accounted for in the analysis of mortality over the duration of 12 months therapy. Also, there was no HAART discontinuation in any of our cohorts which have allowed for the intent-to-treat assumption and reliable interpretations of HAART effect. However, caution should be applied in the generalization of the findings of this study as its small sample size may have had a profound effect on the power to independently detect the significance of some known predictors (HAART regimens, tuberculosis, WHO clinical stage, virological failure, and undernutrition) of mortality in HIV infection at multivariate analysis.

## 6. Conclusions

This study has shown that the initial high mortality seen in the first 6 months of HAART was significantly reduced before the end of 12 months of ART. It suggests a high baseline viral load and severe immunosuppression, as the independent predictors of mortality in the early and late phase of HAART, respectively. This knowledge should allow for a thorough targeted early initiation of HAART for these HIV-infected children who are at a higher risk of dying.

This recommendation would be in conformity with the 90-90-90 targets for identifying HIV-infected children, bringing them to care, initiating ART, and retaining them in care in order to achieve a sustained virologic suppression [60].

In addition, other measures that have been proven to reduce mortality in severely immunocompromised HIV-infected individuals initiating ART in Africa can also reduce further the mortality seen in our setting, and can also be applied [61, 62]. These measures include the use of ready-to-use supplementary food (RUSF) and an enhanced anti-infection prophylaxis (EAIP). The EAIP comprises trimethoprim-sulfamethoxazole plus 12 weeks of isoniazid-pyridoxine, 12 weeks of fluconazole, 5 days of azithromycin, and a single dose of albendazole against mortality from tuberculosis, cryptococcal infection, oral/oesophageal candidiasis, and severe bacterial infections [61, 62].

## Figures and Tables

**Table 1:** Predictors of mortality at 6 months of highly active antiretroviral therapy.

Clinical variables	Mortality	No mortality	Bivariate analysis			Multivariate analysis		
			cHR	95% CI	*p* value	aHR	95% CI	*p* value
*Baseline demography*								
Age (months)								
<12	10 (31.2)	22 (68.8)	274060.24	—	0.840	—	—	—
12–35	16 (28.6)	40 (71.4)	274243.21	—	0.840	—	—	—
36–59	4 (6.1)	62 (93.9)	47987.49	—	0.862	—	—	—
>59 (ref)	0 (0.0)	206 (100.0)	—	—	—	—	—	—
Gender								
Female	12 (7.4)	150 (92.6)	0.806	0.388–1.673	0.562	—	—	—
Male (ref)	18 (9.1)	180 (90.9)	—	—	—	—	—	—
HAART regimens on enrolment								
EFV based	10(21.7)	36 (78.3)	8.515	3.360–21.578	0.001	1.963	0.462–8.332	0.361
ABC/3TC/AZT or D4T	12 (42.9)	16 (57.1)	17.577	7.173–43.069	0.001	2.104	0.580–7.629	0.258
NVP based	8 (2.8)	278 (97.2)	—	—	—	—	—	—
Prevalent TB at enrolment								
Yes	21 (28.8)	52 (71.2)	10.412	4.766–22.745	0.001	1.444	0.420–4.963	0.560
No (ref)	9 (3.1)	276 (93.2)	—	—	—	—	—	—
*Baseline clinical/laboratory findings*								
WHO clinical staging								
3 and 4	28 (12.6)	194 (87.4)	8.702	2.073–36.531	0.003	1.863	0.365–9.508	—
1 and 2 (ref)	2 (1.4)	136 (98.6)	—	—	—	—	—	—
*CD4 count* Severe immunosuppression^#^								
Yes	30 (25.2)	89 (74.8)	218.350	6.322–7541.150	0.003	137813		0.875
No (ref)	0 (0.0)	241 (100)	—	—	—	—	—	—
Viral load (copies/ml)								
≥10,000	29 (13.7)	182 (86.3)	22.217	3.026–163.111	0.002	18.089	2.428–134.77	0.005
<10,000 (ref)	1 (0.7)	148 (99.3)	—	—	—	—	—	—
Anaemia (<8 g/dl)								
Yes	2 (3.2)	60 (96.8)	0.327	0.078–1.374	0.127	—	—	—
No (ref)	28 (9.4)	270 (90.6)	—	—	—	—	—	—
WHZ								
<−2 SD	6 (15.0)	34 (85.0)	0.731	0.299–1.788	4.492	—	—	—
≥−2 SD (ref)	24 (21.1)	90 (78.9)	—	—	—	—	—	—
BMI								
<18.5	0 (0.0)	166 (100.0)	1.00	0.001–1904.991	1.00	—	—	—
≥18.5 (ref)	0 (0.0)	40 (100)	—	—	—	—	—	—

Ref = referenced variable; TB = tuberculosis; cHR = crude hazard ratio; aHR = adjusted hazard ratio; 95% CI = 95% confidence interval; NVP = nevirapine; EFV = efavirenz; LPV/r = lopinavir/ritonavir; ABC = abacavir; 3TC = lamivudine; AZT = zidovudine; d4T = starvudine; HAART = highly active antiretroviral therapy; WHO = World Health Organization; WHZ = weight for health Z score; BMI = body mass index; ^#^2006 WHO age-dependent immunological criteria dichotomized into Yes or No for severe immunosuppression.

**Table 2:** Predictors of mortality at 12 months of highly active antiretroviral therapy.

Clinical variables	Mortality	No mortality	Bivariate analysis			Multivariate analysis		
			cHR	95% CI	*p* value	aHR	95%CI	*p* value
*Baseline demography*								
Age (months)								
<12	0 (0)	22 (100)	0.000	—	0.987	—	—	—
12–35	2 (5.6)	34 (94.4)	1.309	0.278–6.163	0.734	—	—	—
36–59	0 (0)	58 (0)	0.000	—	0.980	—	—	—
>59 (ref)	8 (4.2)	184 (95.8)	—	—	—	—	—	—
Gender								
Female	4 (2.9)	134 (97.1)	0.834	0.235–2.954	0.778	—	—	—
Male (ref)	6 (3.5)	164 (96.5)	—	—	—	—	—	—
HAART regimens at 6 month								
EFV based	0 (0)	48 (100)	0.000	—	0.984	0.024	0.000–576.64	0.470
LPV/r based	6 (13.6)	38 (86.4)	7.975	2.248–28.284	0.001	1379	—	0.952
ABC/3TC/AZT or D4T	0 (0)	2 (100)	0.000	—	0.997	4.143	—	0.987
NVP based	4 (1.9)	210 (98.1)	—	—	—	—	—	—
Incident TB								
Yes	3 (8.3)	33 (91.4)	1.314	0.279–6.190	0.729	—	—	—
No (ref)	7 (2.6)	265 (97.4)	—	—	—	—	—	—
Virological failure within first 6 months								
Yes	6 (13.6)	38 (86.4)	9.892	2.789–35.088	0.001	167.9	—	0.941
No (ref)	4 (1.5)	260 (98.5)	—	—	—	—	—	—
WHO clinical staging								
3 and 4	4 (2.2)	178 (97.8)	0.450	0.127–1.594	0.216	—	—	—
1 and 2 (ref)	6 (4.8)	120 (95.2)	—	—	—	—	—	—
*Laboratory response to HAART at 6 months*								
CD4 count at 6 months Severe immunosuppression^#^								
Yes	6 (8.6)	64 (91.4)	5.40	1.523–19.139	0.009	17.28	3.844–77.700	0.001
No (ref)	4 (1.7)	234 (98.3)	—	—	—	—	—	—
Anaemia (<8 g/dl) at 6 months								
Yes	2 (5.0)	38 (95.0)	1.631	0.346–7.683	0.536	—	—	—
No (ref)	8 (3.0)	260 (97.0)	—	—	—	—	—	—
*Anthropometric response to HAART at 6 months*								
WHZ at 6 months								
<−2 SD	0 (0)	2 (100)	0.000	—	0.987	—	—	—
≥−2 SD (ref)	2 (1.8)	112 (98.2)	—	—	—	—	—	—
BMI at 6 months								
<18.5	4 (2.4)	162 (97.6)	0.153	0.038–0.611	0.008	0.001	0.000–6.223	0.120
≥18.5 (ref)	4 (15.4)	22 (84.6)	—	—	—	—	—	—

Ref = referenced variable; TB = tuberculosis; cHR = crude hazard ratio; aHR = adjusted hazard ratio; 95% CI = 95% confidence interval; NVP = nevirapine; EFV = efavirenz; LPV/r = lopinavir/ritonavir; ABC = abacavir; 3TC = lamivudine; AZT = zidovudine; d4T = starvudine; HAART = highly active antiretroviral therapy; WHO = World Health Organization; WHZ = weight for health Z-score; BMI = body mass index; ^#^2006 WHO age-dependent immunological criteria dichotomized into Yes or No for severe immunosuppression.

**Table 3:** Causes of deaths among the HIV-infected children within 12 months of highly active antiretroviral therapy.

Causes of death	Number (%)
*Within the first 6 months*	
Tuberculosis^*∗*^	21 (70)
Unknown^#^	4 (13.3)
Sepsis/undernutrition	3 (10)
Severe pneumonia	2 (6.7)
Total	30 (100)
*At the later 6 months of follow-up*	
Severe pneumonia	4 (40)
Unknown^#^	3 (30)
Tuberculosis^§^	3 (30)
Total	10 (100)

^*∗*^The 21 children had prevalent tuberculosis with deaths resulting from 11 of those with pulmonary tuberculosis, 7 with tuberculosis meningitis/miliary tuberculosis, 2 with gastrointestinal tuberculosis, and 1 with tuberculosis adenitis; ^§^2 deaths from tuberculosis meningitis/miliary tuberculosis and 1 death from pulmonary tuberculosis; ^#^caregivers could only give history of febrile illness.

## Data Availability

The caregivers and parents of children and the children included in this evaluation had not consented to sharing of their patient-level data, and for us to do so would be a violation of their privacy. As such, due to issues with patient confidentiality, the patient-level data on which the analyses were based cannot be posted in a public forum.
